# Letter to the Editor—American Journal of Men’s Health: Prostatitis

**DOI:** 10.1177/15579883211019868

**Published:** 2021-05-26

**Authors:** Ericka Johnson

**Affiliations:** 1 Professor of gender and society, Linköping University, Linkoping, Sweden

Dear Editors,

I would like to respond to the recent review, “Chronic Prostatitis/Chronic Pelvic Pain Syndrome” (Zhang et al 2020). Firstly, I applaud the thoroughness of this study. As the PI of a medical humanities research project on the prostate, with prostatitis as one of our research topics, I feel that this diagnosis is, as the authors point out, a very complex collection of symptoms which causes frustration for both urologists and patients. The authors cover this complexity with aplomb.

As a medical sociologist, I would also like to applaud the authors’ recognition of social factors related to the diagnosis/syndrome. I, too, have found that there are a multitude of social and relational aspects that both patients and medical professionals deal with, and recognition of this from urologists highlights the complex entanglement of bio-medical and social aspects of life. In particular, research shows that relationships and lifestyle aspects of the man’s situation—a partner’s presence and support, the roles of jobs, sports, and social activities—both impact and are impacted by prostatitis/chronic pelvic pain. This has been particularly emphasized in my research which engaged patients and medical professionals from outside of urology, including sexual therapists, psychologists and urotherapists with physiotherapy backgrounds (Johnson 2021). It is reassuring to see that their important professional insights are in line with insights reported from within urology.

However, one point which my medical humanities research has indicated, and which was not covered in the review, is the role of medical care structures (disciplines and specialities) in the production of a prostatitis diagnosis. As it stands now, in many national healthcare systems a patient who presents with urination issues or diffuse pain in the lower abdomen *and who has a prostate* will most likely first seek care with their general practitioner and then be referred to a urologist. A patient who presents with urination issues or diffuse pain in the lower abdomen and *who has a uterus* will most likely be referred to a gynaecologist. This leads me to ask: does our current division of specialist care into a binary system grounded on reproductive organs (gynaecology and urology) lead to cases of chronic pelvic pain in bodies with a prostate being misdiagnosed as prostatitis? Are perhaps many of these cases related to the pelvic muscle structures which could be better treated outside of urology? And if so, is the prostate becoming a scapegoat for diffuse symptoms and complaints which, within a different medical care structure, could be identified as related to “human” rather than “male” anatomical structures?

Kind regards,

(removed for review)
